# Time-varying serum albumin levels and all-cause mortality in prevalent peritoneal dialysis patients: a 5-year observational study

**DOI:** 10.1186/s12882-019-1433-8

**Published:** 2019-07-10

**Authors:** Na Hao, Ben-Chung Cheng, Hong-Tao Yang, Chien-Hsing Wu, Yang-Yang Lei, Mei-Chen Chao, Pei-Ying Wang, Li-Chueh Kuo, Sin-Hua Moi, Cheng-Hong Yang, Jin-Bor Chen

**Affiliations:** 1 Division of Nephrology, First Teaching Hospital of Tianjin University of Traditional Chinese Medicine, 88 Chang Ling Rd, Xi Qing District, Tianjin, China; 2 Division of Nephrology, Department of Internal Medicine, Kaohsiung Chang Gung Memorial Hospital and Chang Gung University College of Medicine, No.123 Dapi Road, Niaosong District, Kaohsiung City, 83301 Taiwan; 3 Peritoneal Dialysis Center, Division of Nephrology, Kaohsiung Chang Gung Memorial Hospital and Chang Gung University College of Medicine, No.123 Dapi Road, Niaosong District, Kaohsiung City, 83301 Taiwan; 4 Department of Electronic Engineering, National Kaohsiung University of Science and Technology, No. 415 Jiangong Road, Sanmin District, Kaohsiung City, 80778 Taiwan; 5 Ph. D. Program in Biomedical Engineering, Kaohsiung Medical University, No. 100, Shiquan 1st Road, Sanmin District, Kaohsiung City, 807 Taiwan

**Keywords:** Albumin, Mortality, Peritoneal dialysis

## Abstract

**Background:**

In this study, we investigated the association of time-varying serum albumin levels with mortality over a 5-year period in one cohort of patients undergoing long-term peritoneal dialysis (PD) therapy.

**Methods:**

The participants in this study enrolled 302 patients who underwent long-term PD at a single PD center in Taiwan. We reviewed medical records from 2011 to 2015 retrospectively. Time-averaged albumin level and serum albumin reach rate (defined as the percentage of serum albumin measurements that reached ≥3.5 g/dL) were applied as the predictor variables in the first 2 years (2011–2012). All-cause mortality was used as the outcome variable in the subsequent 3 years (2013–2015). Hazard function of all-cause mortality in the study participants was examined by using Cox proportional hazard regression models .

**Results:**

Patients with different albumin reach rates (75–< 100%, 50–< 75%, 1–< 50%) did not exhibit a significantly increased risk for all-cause mortality. Patients with a 0% albumin reach rate exhibited a significantly increased risk for all-cause mortality (hazard ratio [HR] 7.59, 95% confidence interval [CI], 2.38–24.21) by fully adjusted analysis. Patients with time-averaged albumin levels of < 3.5 g/dL (HR 15.49, 95% CI 1.74–137.72) exhibited a higher risk for all-cause mortality than those with serum albumin levels ≥4.0 g/dL.

**Conclusions:**

This study demonstrated that higher serum albumin reach rates and higher time-averaged serum albumin levels are associated with a lower mortality rate over a 5-year period among patients undergoing long-term PD.

## Background

The number of patients undergoing peritoneal dialysis (PD) is increasing worldwide. It is estimated that there are more than 272,000 PD patients, accounting for 11% of the dialysis population [1]. Despite advances in dialysis technology, dialysis patients still develop multisystem metabolic disorders, leading to high and accelerated mortality rates [2]. Among the metabolic disorders, protein-energy wasting is an emerging concern for nephrologists. There has been a consensus that nutritional intervention improves nutritional parameters and survival rates [3].

Serum albumin level is an important observation index for assessing nutritional status, and its effect on mortality in dialysis patients has been examined [4–9]. Several clinical circumstances have been reported to result in low serum albumin levels, including inadequate dialysis [10], fluid overload, inflammatory and infectious diseases [11, 12], co-morbidities [13], and taste changes [14]. Low serum albumin levels are associated with high mortality rates in dialysis patients. One study found that a persistently low serum albumin level for 6 months was associated with all-cause and cardiovascular mortalities in the subsequent months [8]. However, this study has scarce data on the time-varying levels of serum albumin and its association with long-term survival in dialysis patients.

The aim of this cohort study was to investigate the long-term effect of serum albumin levels on the survival of prevalent PD patients. We measured 2 predictor variables, target albumin reach rates and time-averaged albumin levels, over 2 years to predict the subsequent 3-year all-cause mortality.

## Methods

### Participants

The records of 555 prevalent PD patients who were tracked from 2011 to 2015 at Kaohsiung Chang Gung Memorial Hospital in Taiwan were reviewed. We excluded 253 patients because of incomplete demographic and laboratory data, and 302 patients were considered eligible for the survival analysis. The exclusion criteria were as follows: (1) age < 18 years, (2) incomplete clinical information, and (3) transfer to other hospital or kidney transplantation. The requirement for patient consent was waived by the Institutional Review Board of Chang Gung Memorial Hospital (document no. 201800595B0), in accordance with the principles of the Declaration of Helsinki. In keeping with the retrospective data review regulations of the Committee on Human Research of Kaohsiung Chang Gung Memorial Hospital, signed informed consent from the study participants was not required.

### Laboratory measurements

The participants enrolled in the study underwent monthly hematological and biochemical examinations in the outpatient clinic. Baseline values that were collected and measured in January 2011 included hemoglobin (Hb), albumin, potassium (K), corrected serum calcium (Ca), phosphate (P), intact parathyroid hormone (iPTH), alkaline phosphate (ALP), cholesterol, triglyceride, 24-h urine volume, total Kt/V, weekly urine creatinine clearance rate (Ccr), total weekly Ccr, and protein catabolic rate normalized using actual body weight. The formula for calculating the protein catabolic rate was 10.76 × {[(UV × U_BUN_ + DV × D_BUN_)/1440 + 1.46]/BSA} × 1.73, where UV is the urine volume (L), DV is the dialysate volume (L), U_BUN_ is the urine blood urea nitrogen (BUN), D_BUN_ is the dialysate BUN, and BSA is the body surface area. All blood samples were analyzed using commercial kits and an auto-analyzer (Hitachi 7600–210; Hitachi Ltd., Tokyo, Japan). Albumin level was measured using the bromocresol green method; the normal range in the hospital was 3.5–5.2 g/dL. iPTH was measured using a chemiluminescence immunoassay (Siemens Healthcare Diagnostics Inc., Tarry Town, NY, USA). The parameters of standard peritoneal equilibration tests measured in January 2011 were collected as baseline data.

### Stratified serum albumin levels

We used the time-averaged albumin level and serum albumin reach rate (target level: 3.5 g/dL) in 2011–2012 as the predictor variables. All-cause mortality in 2013–2015 was used as the outcome variable. The time-averaged albumin level was the average of the 24-month serum albumin level in 2011–2012. We defined serum albumin reach rate in the first 2 years (2011–2012) as the percentage of albumin measurements that reached ≥3.5 g/dL. Two serum albumin stratifications were used for analyses. We stratified the albumin reach rate into 5 groups depending on the serum albumin levels that reached 3.5 g/dL among the 24-month measurements. A 100% albumin reach rate indicated that all 24 monthly serum albumin measurements had reached 3.5 g/dL, and 0% indicated that none of the 24 monthly serum albumin measurements had reached 3.5 g/dL. Subsequently, we divided the participants into 2 groups according to the percentage of monthly serum albumin measurements that reached ≥3.5 g/dL over the first 2-year (2011–2012) follow-up period. Patients with ≥75% serum albumin measurements reaching ≥3.5 g/dL were classified as the higher rate group, whereas those with < 75% serum albumin measurements reaching ≥3.5 g/dL were indicated as the lower rate group.

### Outcome measures

The outcome measures included the association between the serum albumin reach rate and time-averaged albumin levels in the first 2 years, with the subsequent 3-year all-cause mortality.

### Statistical analysis

The baseline characteristics and clinical variables of the study population were grouped according to 24 months albumin rate reached at 3.5 g/dL and summarized as frequency (percentage) or mean (standard deviation). The difference between groups was estimated using independent two-sample t-test, chi-squared test, or Fisher’s exact test. The Cox proportional hazard regression model was used to determine the association between different albumin categories by using 24-month serum albumin measurements and all-cause mortality. A *P*-value of < 0.05 was considered statistically significant. Stata version 11.0 (StataCorp. 2009. Stata Statistical Software: Release 11. College Station, TX: StataCorp LP.) was used for all statistical analyses.

## Results

A total of 302 patients were analyzed. During the 5-year period, 205 patients had high albumin levels, whereas 97 patients had low albumin levels. Older patients had lower serum albumin reach rates. Data of blood parameters showed that patients with lower albumin reach rates exhibited significantly lower values of Hb, albumin, P, and dialysis Kt/V than did those with higher albumin reach rates. However, ALP was significantly higher in patients with lower albumin reach rates (Table 1).

**Table 1:** Baseline characteristics and clinical features of the study population according to different albumin levels (*N* = 302)

Variable	24 months albumin rate reached 3.5(g/dL)				*P*-value
	Higher rate (*n* = 205)		Lower rate (*n* = 97)		
	Mean	SD	Mean	SD	
Dialysis vintage (years)	8.07	2.95	7.58	3.49	0.207
Age (years)	50.22	12.65	54.75	14.38	0.006
Gender (n, %)					0.241
Male	95	46.34	38	39.18	
Female	110	53.66	59	60.82	
Diabetes Mellitus (n, %)	31	15.12	20	20.62	0.234
Blood analysis					
Hb (g/dL)	10.70	1.41	10.06	1.43	< 0.001
Albumin (g/dL)	3.97	0.30	3.45	0.40	< 0.001
K (meq/L)	4.30	0.75	4.18	0.71	0.181
Ca (mg/dL)	9.29	0.75	9.40	0.85	0.270
P (mg/dL)	5.46	1.36	5.12	1.08	0.033
iPTH (pg/mL)^a^	100	(41.6–377)	93	(31.6–203)	0.594
ALP (U/L)^a^	88	(67–116)	99	(73–156)	0.018
Cholesterol (mg/dL)^a^	181	(161–205)	178	(158–197)	0.157
Triglyceride (mg/dL)^a^	133	(91–196)	115	(82–193)	0.213
Urine volume 24HR (mL)^a^	600	(400–920)	600	(400–720)	0.252
Kt/V	2.14	0.36	2.00	0.40	0.004
Weekly urine Ccr^a^	14.43	(9.92–20.73)	14.43	(13.93–14.425)	0.499
Weekly total Ccr^a^	59.01	(50.74–69.1)	59.01	(50.94–65.25)	0.475
nPCR (g/kg/day)	1.08	0.25	1.06	0.23	0.476

Patients aged < 18 years (*n* = 2) were excluded

^a^Median (interquartile range)

*P*-values of categorical variables were estimated using the χ2 test, and those for continuous variables were estimated using the 2-sample t-test

*Hb* hemoglobin, *K* potassium, *Ca* corrected serum calcium, *P* phosphate, *iPTH* intact parathyroid hormone, *ALP* alkaline phosphate, *Ccr* creatinine clearance rate, *nPCR* normalized protein catabolic rate

Table 2 shows the distribution of the study population in different albumin classes (measured using 2-year serum albumin levels) and mortality rates based on stratified serum albumin levels. The trend of all-cause mortality showed a parallel increase with decreased serum albumin reach rates. A similar trend was observed in time-averaged serum albumin levels.

**Table 2:** Different albumin categories based on 24-month serum albumin measurements in the study population

Albumin category	Total		All-cause mortality	
	n	%	n ^a^	%
Albumin rate reached 3.5(g/dL)				
100	115	38.08	8	6.96
75 to < 100	90	29.80	8	8.89
50 to < 75	35	11.59	6	17.14
1 to < 50	40	13.25	7	17.50
0	22	7.28	11	50.00
Albumin rate reached 3.5(g/dL)				
Higher rate (75 to 100)	205	67.88	16	7.80
Lower rate (0 to < 75)	97	32.12	24	24.74
Time-averaged serum albumin				
≥ 4	47	15.56	1	2.13
3.5 to < 4	181	59.93	20	11.05
< 3.5	74	24.50	19	25.68

^a^ (Number of all-cause mortalities/total number in each category) × 100%

Table 3 shows the association between different albumin stratification levels (measured using the serum albumin levels for the first 24 months) and all-cause mortality over the subsequent 3 years based on Cox regression analysis. Adjusted analysis showed that the risk of all-cause mortality in the participants increased parallel to low albumin reach rates. Participants who had a 0% albumin reach rate had the highest statistically significant risk for all-cause mortality (hazard ratio [HR] 15.49, 95% confidence interval [CI] 1.74–137.72). Stratified time-averaged serum albumin levels showed a similar trend. Participants with time-averaged albumin levels < 3.5 g/dL exhibited a 15.49-fold increased rate of all-cause mortality as compared with those with the reference level (≥4.0 g/dL).

**Table 3:** Associations between different albumin categories based on 24-month serum albumin measurements and all-cause mortality in the study population

Albumin category	All-cause mortality			
	Deaths (n)	Unadjusted	Adjusted (demographic)^a^	Adjusted^b^
		HR (95% CI)	HR (95% CI)	HR (95% CI)
Albumin rate reached 3.5(g/dL)				
100	8	Reference	Reference	Reference
75 to < 100	8	1.29 (0.48–3.43)	1.2 (0.45–3.23)	0.9 (0.28–2.84)
50 to < 75	6	2.61 (0.9–7.51)	2.32 (0.79–6.76)	2.56 (0.8–8.24)
1 to < 50	7	2.66 (0.97–7.35)	2.33 (0.82–6.6)	2.85 (0.85–9.6)
0	11	11.04 (4.43–27.5)	5.63 (1.97–16.09)	7.59 (2.38–24.21)
Albumin rate reached 3.5(g/dL)				
Higher rate (75 to 100)	16	Reference	Reference	Reference
Lower rate (0 to < 75)	24	3.59 (1.91–6.76)	2.66 (1.35–5.24)	3.57 (1.66–7.65)
Time-averaged serum albumin				
≥ 4	1	Reference	Reference	Reference
3.5 to < 4	20	5.33 (0.72–39.74)	4.11 (0.55–30.8)	5.81 (0.72–47.12)
< 3.5	19	14.12 (1.89–105.46)	6.95 (0.91–53.27)	15.49 (1.74–137.72)

Univariate and multivariate Cox proportional hazard regression models

Adjusted (demographic)^a^, adjusted for demographic variables including dialysis vintage, age, sex, and diabetes mellitus

Adjusted^b^, adjusted for all demographic and laboratory variables

*HR* hazard ratio, *CI* confidence interval

## Discussion

Serum albumin is commonly considered to be a strong predictor of mortality in dialysis patients [5, 6, 9, 15–17]. Prior studies considered time-varying changes in serum albumin levels in examining the relationship of serum albumin level with mortality in dialysis patients [9, 17]. In a 19-month study, investigators found that serum albumin levels measured before PD initiation was better at predicting mortality than albumin levels measured at PD initiation [17]. Our previous study demonstrated that hemodialysis patients with lower time-averaged serum albumin levels had higher mortality rates over 5 years [9]. This study investigated the relationship of target serum albumin reach rates and time-averaged albumin levels with mortality in PD patients over a 5-year period. PD patients with lower target serum albumin reach rates and lower time-averaged albumin levels demonstrated a higher all-cause mortality. The results demonstrated the long-term effect of serum albumin levels on the mortality risk of PD patients and showed that a sustained serum albumin level is crucial for maintaining survival benefit in prevalent PD patients.

In this study, we set the minimum standard for serum albumin level at 3.5 g/dL. This criterion was based on the lower normal limits of our laboratory tests and the standard of the National Health Insurance Authority of Taiwan (http://sc-drtw/news/104/010603.htm; http://sc-drtw/news/104 /01/01060301.pdf). This cutoff level is lower than the recommended level of 3.8 g/dL by the International Society of Renal Nutrition and Metabolism [3]. We acknowledge that the lower target serum albumin level used in this study may underestimate the percentage of lower serum albumin level in our cohort. However, because our study uses time-varying serum albumin levels, we hypothesized that the target serum level set in our study could not interfere with the trend of albumin changes during the observation period. Nevertheless, further studies are needed to validate the optimal serum albumin level for PD patients.

In this study, the Kt/V values of participants with high albumin reach rates were significantly higher than those with low albumin reach rates. Multivariate regression analysis showed that baseline serum albumin levels had a positive correlation with total Kt/V and a negative correlation with total weekly Ccr. We also found that weekly urine Ccr did not have a statistically significant correlation with the baseline serum albumin level. It is generally believed that adequate dialysis has a significant impact on the outcome of PD patients [18]. Our previous study demonstrated that insufficient dialysis could lead to worsening nutritional status [19]. Minimum dialysis adequacy indices have been recommended to maintain optimal dietary nutrient intake to avoid protein-energy wasting in dialysis patients [3, 20]. However, studies that directly examine the effect of increased dialysis dose on nutritional parameters in dialysis patients are scarce. Our study has a cross-sectional design; hence, we could not examine the cause-effect relationship between dialysis adequacy and albumin level. We believe that it is worthy to conduct a study investigating the association between dialysis dose and nutritional parameters in PD patients in the future.

The present study has several limitations. First, its cross-sectional retrospective design does not allow for examining the effect of immortal time. Therefore, we used the serum albumin levels in the first 2 years to examine their effect on mortality in the subsequent 3 years. A similar method was also applied by other relevant studies [8, 9, 21]. Second, our study did not include other possible factors affecting nutritional parameters, such as economic and social status, comorbidities, hospitalizations, and dialysis protocols. Third, the sample size was very small resulting in a wide confidence interval in the significance findings (HR 15.49, 95% CI 1.74–137.72) and reduce the reliability of current findings. Hence, the results should be validated in a larger cohort study. On the other hand, this study has some strengths. First, our study is the first to use 2-year target serum albumin reach rate and time-average albumin level to examine the correlation with mortality in the next 3 years in prevalent PD patients. Second, the observation period was 5 years. Therefore, the long-term trend in serum albumin levels in prevalent PD patients was observed, and examination of the relationship between time-varying serum albumin levels and mortality was possible.

## Conclusions

This study demonstrated that target serum albumin reach rate and time-averaged albumin levels were associated with all-cause mortality in prevalent PD patients. This result emphasizes the importance of serum albumin level as a surrogate predictor for mortality in PD patients.

## Data Availability

All data supporting the study are presented in the manuscript or are available upon request from the corresponding author of this manuscript (Jin-Bor Chen, e-mail address: chenjb1019@gmail.com).

## Abbreviations

ALP: Alkaline phosphate
Ca: Corrected serum calcium
Ccr: Creatinine clearance rate
CI: Confidence interval
Hb: Hemoglobin
HR: Hazard ratio
iPTH: Intact parathyroid hormone
K: Potassium
nPCR: Normalized protein catabolic rate
P: Phosphate
PD: Peritoneal dialysis
